# Predicting Potential Endocrine Disrupting Chemicals Binding to Estrogen Receptor α (ERα) Using a Pipeline Combining Structure-Based and Ligand-Based in Silico Methods

**DOI:** 10.3390/ijms22062846

**Published:** 2021-03-11

**Authors:** Asma Sellami, Matthieu Montes, Nathalie Lagarde

**Affiliations:** Laboratoire GBCM, EA 7528, Conservatoire National des Arts et Métiers, Hésam Université, 2 rue Conté, F-75003 Paris, France; asma.sellami@lecnam.net

**Keywords:** nuclear receptors, ERα, endocrine disrupting chemicals, docking, pharmacophores, virtual screening

## Abstract

The estrogen receptors α (ERα) are transcription factors involved in several physiological processes belonging to the nuclear receptors (NRs) protein family. Besides the endogenous ligands, several other chemicals are able to bind to those receptors. Among them are endocrine disrupting chemicals (EDCs) that can trigger toxicological pathways. Many studies have focused on predicting EDCs based on their ability to bind NRs; mainly, estrogen receptors (ER), thyroid hormones receptors (TR), androgen receptors (AR), glucocorticoid receptors (GR), and peroxisome proliferator-activated receptors gamma (PPARγ). In this work, we suggest a pipeline designed for the prediction of ERα binding activity. The flagged compounds can be further explored using experimental techniques to assess their potential to be EDCs. The pipeline is a combination of structure based (docking and pharmacophore models) and ligand based (pharmacophore models) methods. The models have been constructed using the Environmental Protection Agency (EPA) data encompassing a large number of structurally diverse compounds. A validation step was then achieved using two external databases: the NR-DBIND (Nuclear Receptors DataBase Including Negative Data) and the EADB (Estrogenic Activity DataBase). Different combination protocols were explored. Results showed that the combination of models performed better than each model taken individually. The consensus protocol that reached values of 0.81 and 0.54 for sensitivity and specificity, respectively, was the best suited for our toxicological study. Insights and recommendations were drawn to alleviate the screening quality of other projects focusing on ERα binding predictions.

## 1. Introduction

Estrogens are hormones involved in many physiological processes such as growth, development, the female reproductive system, and homeostasis [1]. They can exert their activity through binding to particular transcription factors: the estrogen receptors (ER). As members of the nuclear receptor protein family (NRs), ER are composed of three functional domains, the NH2-terminal domain (NTD), the DNA-binding domain (DBD), and the COOH-terminal ligand-binding domain (LBD) [2]. Two isoforms of the receptor exist, ERα and ERβ. Both isoforms share a high degree of sequence identity within their LBDs and exhibit similar affinities for the main endogenous ligand, 17β-estradiol [3], but different affinities for other compounds, given that each subtype displays a unique role in estrogenic activity in vivo. Since its discovery [4], several therapeutic applications have emerged for ERα ligands, in particular in breast cancer therapies [5,6]. Consequently, a large number of small molecules were developed with the purpose of ERα activity modulation. However, some compounds belonging to a particular category of exogenous molecules called endocrine disrupting chemicals (EDCs) are also able to bind to ERα [7]. 

EDCs have the ability to penetrate the body through ingestion, inhalation, or skin and to mimic the endogenous hormones, leading to the disruption of the endocrine system in both human and animal species. The first reported EDCs harmful effects were related to estrogens [8] such as breast cancer, endometriosis, fertility problems, and learning disability. EDCs are now considered a public health threat [9,10,11], as human exposure to these compounds can increase the risk of impairment of several biological functions such as the reproductive [12], cognitive [13], and metabolic [14] functions (for a review of associations between EDC exposures and risk to diseases, see Table 1 in [15]). However, the knowledge about possible adverse effects of EDCs is still incomplete and numerous studies have focused on better understanding their mechanism of action. 

EDCs have been shown to act through direct or indirect mechanisms. In the direct mechanism, EDCs directly bind to a receptor of the NRs family (estrogen receptors ER, thyroid hormones receptors TR, androgen receptors AR, glucocorticoid receptors GR, and peroxisome proliferator-activated receptors gamma PPARγ) or the aryl hydrocarbon receptor, leading to activation or inhibition of its signaling pathway. In the indirect mechanism, EDCs affect other transcription factors or hormone metabolism through interaction with components of the hormone signaling pathway, stimulation or inhibition of endogenous hormones biosynthesis, binding to circulating hormone-binding protein, stimulation or inhibition of hormone-binding protein synthesis or degradation, stimulation or inhibition of hormone receptor expression [16,17]. Other potential targets of EDCs include the membrane-associated NRs and the G protein-coupled receptor GPR30/G protein-coupled estrogen receptor [18]. Experimental campaigns are conducted to identify potential EDCs and better understand their mechanism of action. 

With the large and increasing number of compounds suspected to be EDCs, an intermediate step is needed to prioritize or reduce the number of compounds to be assessed. Several in silico methods are providing prediction and estimation of the potential endocrine disrupting activity of chemicals [19,20,21,22]. The majority of the in silico studies dedicated to EDCs focused on the direct mechanism. These studies are dedicated to NR binding prediction and most studies available are related to ERα [21,22,23,24,25]. These studies considered that a compound predicted to be able to bind to ERα can be a potential EDC that should be further investigated experimentally. In silico predictions of EDCs are mostly done through QSAR models and machine learning methods that provide a quantitative estimation of the binding affinity or a classification of the potential hazard. Docking methods are also used but to a lesser extent despite the advantage of providing insights on the molecular mechanism of binding [19].

In the present work, we designed a pipeline for the prediction of compounds binding to ERα. These flagged compounds can be further explored using experimental techniques to assess their potential to be EDCs. This pipeline combines structure-based (SB) and ligand-based (LB) methods, i.e., docking, SB, and LB pharmacophore models. To select the optimal docking protocol for ERα binding (B) compounds prediction, the performance of different docking software was evaluated and docking scores thresholds were defined. A combination of 26 pharmacophore models was designed to guarantee a maximum coverage of the chemical space of ERα B compounds. Individual performances of LB and SB models to discriminate between B and non-binding (NB) compounds were evaluated. Finally, different combination approaches were also explored to define the best protocol for the prediction of ERα binding potential. We conclude our work with recommendations for future ERα (and other NRs) binding prediction studies.

## 2. Results

### 2.1. Compounds and Database Preparation

#### 2.1.1. Database Preparation

**Figure 1:**
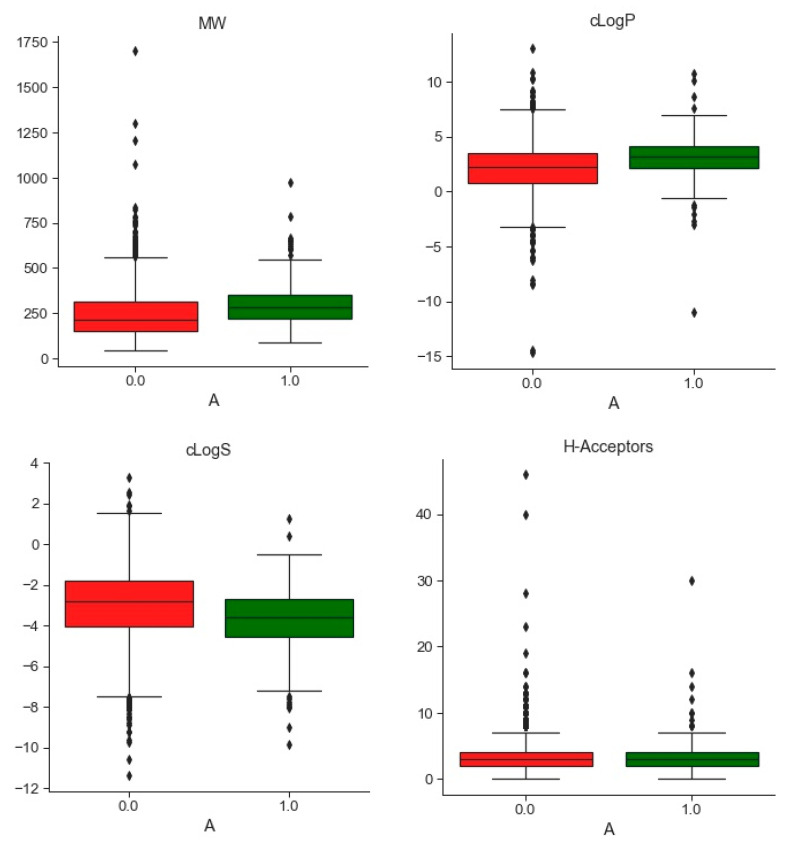

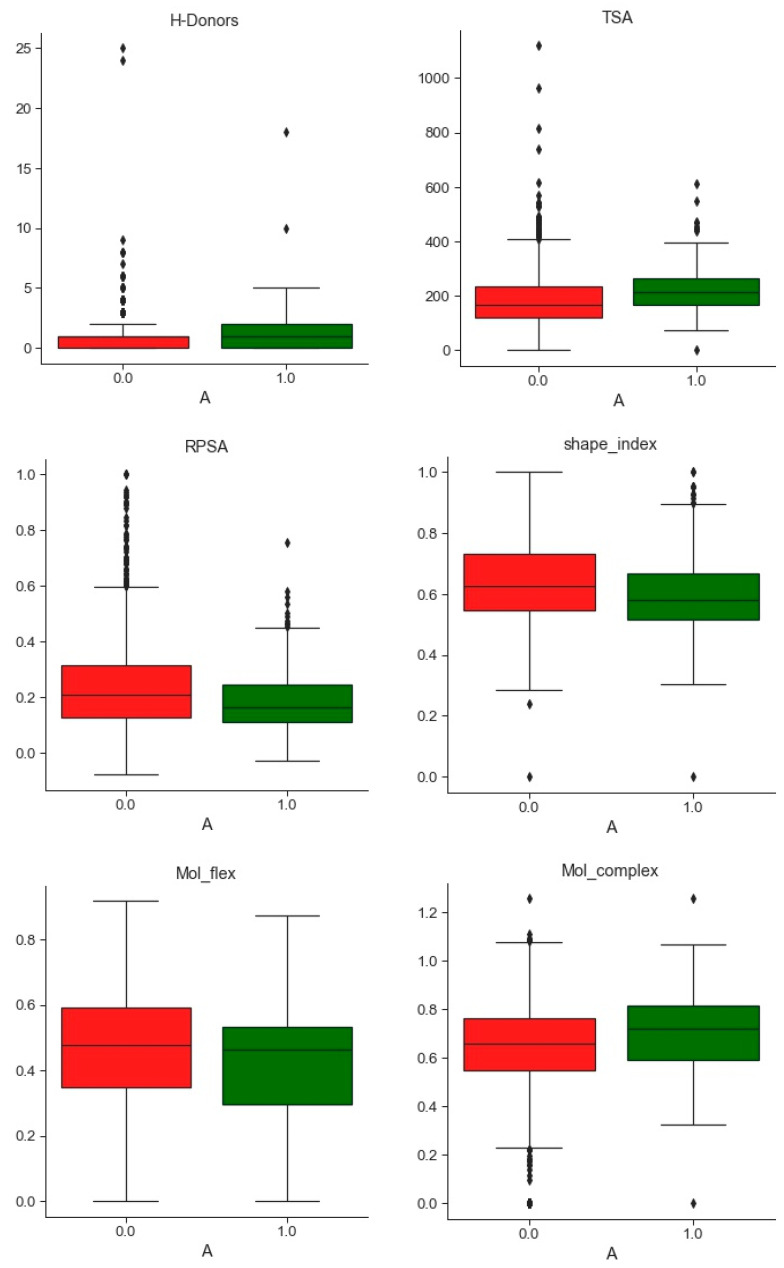

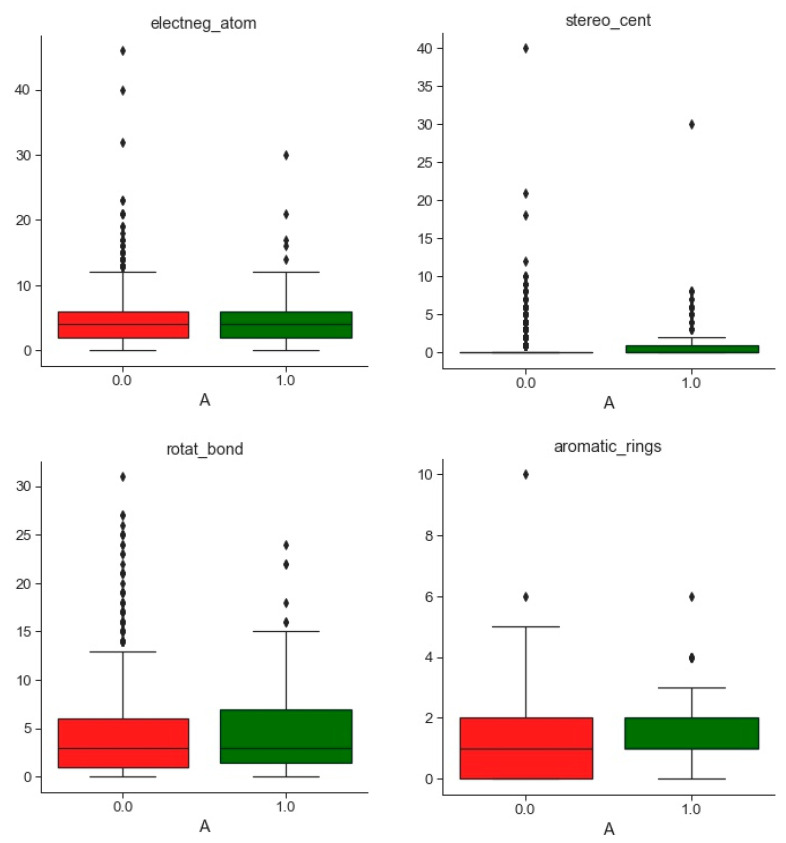

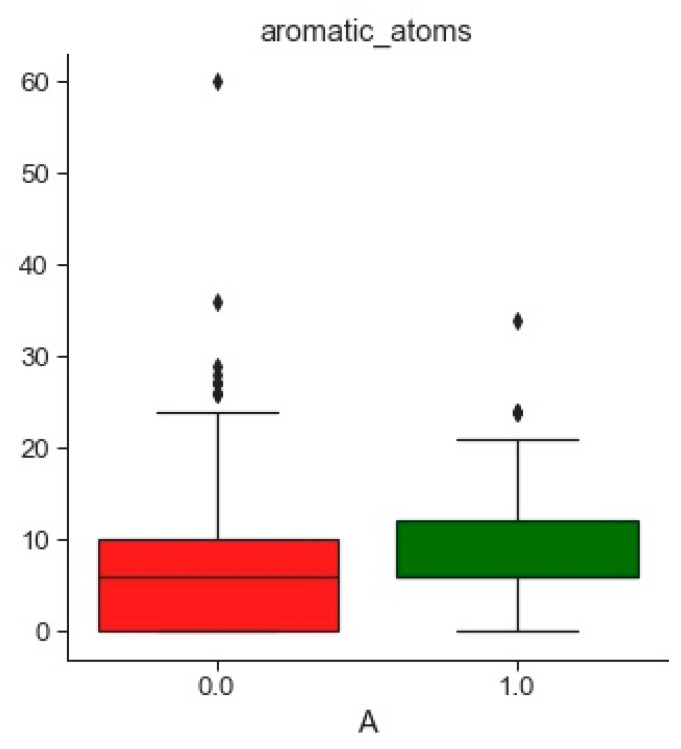
Boxplots representing the distribution of physiochemical descriptors computed with Datawarrior [26] for binding compounds (B) in green and non-binding (NB) compounds in red.

After filtering and cleaning, the Environmental Protection Agency (EPA) database is a collection of 2442 chemical compounds experimentally tested for ERα binding comprising 2219 non-binding (NB) compounds and 223 binding (B) compounds (see Material and Methods section). The distribution of the physiochemical and constitutional descriptors of B and NB compounds is represented in Figure 1.

Two external validation sets were used, i.e., the NR-DBIND (Nuclear Receptors DataBase Including Negative Data) ERα set that comprises 732 compounds, divided into 554 B compounds and 178 NB compounds, and the EADB (Estrogenic Activity DataBase) set comprising 131 B compounds and 101 NB compounds for a total of 232 molecules. Distributions of the 15 constitutional, physiochemical, and molecular descriptors for each dataset are presented in Supplementary Figures S1 and S2.

#### 2.1.2. Databases Comparison

**Figure 2:**
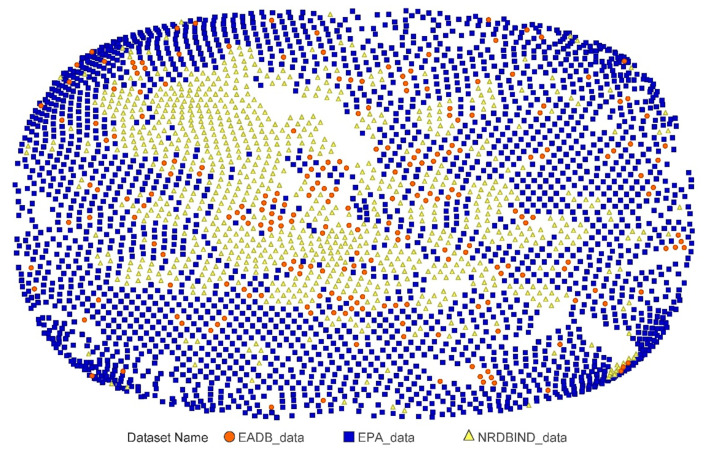
Structure Activity Landscape Index (SALI) maps for all three databases (B and NB compounds): Environmental Protection Agency (EPA) (blue), Nuclear Receptors DataBase Including Negative Data (NR-DBIND) (yellow) and Estrogenic Activity DataBase (EADB) (orange).

Pairwise similarities were calculated using the Tanimoto coefficient (Tc) between each pair of topological fingerprints for: 1.) the EPA database and the NR-DBIND and 2.) the EPA and the EADB (see Figure S3A). The analysis of similarity values shows that the Tc are globally very low with a mean of 0.181 for the pairing with NR-DBIND and 0.174 for the pairing with EADB. Only 2% and 0.6% of the total calculated Tc for EADB and NR-DBIND, respectively (as shown in Figure S3B), are higher than 0.5. Finally, the chemical space of the three databases was mapped using a SALI (Structure Activity Landscape Index) map for the whole databases (Figure 2). The map illustrates that all three databases share the same chemical space.

### 2.2. Docking

#### 2.2.1. Docking Outcome

In order to determine the optimal protocol for discriminating ERα B from NB compounds, 7 molecular docking tools (smina-vina, smina-vinardo, smina-dkoes_scoring, smina-adt4, Protein–Ligand ANT System (PLANTS), and Surflex-dock) were explored using 2 approaches: single structure docking and ensemble docking. Docking performance in predicting B compounds was evaluated using the area under the ROC (Receiver Operating Characteristic) curve (AUC) values (Table 1). For the single structure docking approach, mean AUC are comprised between 0.576 for Surflex-dock (with the largest standard deviation between AUCs) and 0.704 for both smina-dkoes (with the smallest standard-deviation between AUCs) and smina-vinardo. The best performance is obtained using smina with the scoring function dkoes for the 1qku structure with an AUC of 0.708. For all the scoring functions, the structure associated with the best performance displays an agonist-bound conformation.

For the ensemble docking approach, all ensemble sizes, from 2 to 7 structures, were tested but no amelioration in the AUC values was observed with ensembles of more than 3 structures. Table 1 summarizes the results obtained for both single structure and ensemble docking approaches for ensembles of 2 and 3 structures (results for the ensembles of size superior to 3 are presented in Supplementary Table S1). The best mean AUC (0.703) and max AUC (0.710) values are associated with the smina_dkoes scoring function for ensemble of 2 and 3 structures, respectively. The lowest mean AUC (0.594) and max AUC (0.616) were obtained for an ensemble of 2 structures using Surflex-dock.

No significant improvement was observed between single structure and ensemble docking approaches. This is particularly true for both smina-dkoes and PLANTS, for which the best AUC obtained using the ensemble docking approach is almost equal to those obtained with single structure docking. It is to note that for all six scoring functions, the structure associated with the best AUC performance for single structure docking is always present in the best ensemble of 2 and 3 structures.

#### 2.2.2. Predictiveness Curve

Predictiveness curve (PC) was used to define docking score thresholds (TH) associated with a high P(active), i.e., the probability of having active compounds in the screened fraction. For each scoring function and for both docking approaches, i.e., single structure and ensemble docking, TH associated with the highest P(active) were defined. For these TH, sensitivity and specificity values were also deduced. The highest P(active) value is the one of smina-dkoes (~0.3) followed closely by PLANTS (see Table S2). However, these values of P(active)max are associated with a low hit rate. As presented in Table S3, the highest P(active)max is associated with a TH of −10 using smina-dkoes and yields a low hit rate (14 hits out of 2442 compound at start). The same tendency is observed for PLANTS for which the screened subset with the highest probability of activity encompasses few molecules: 5 hits in total without any B among them. Thus, we chose to explore TH associated with various sensitivity levels. Table 2 displays the performances for various sensitivity values for both scoring functions smina_dkoes and PLANTS and for both single structure and ensemble docking approaches. The P(active) and enrichment factor (EF) deducted for these TH yielded better results for smina-dkoes than PLANTS. Regardless, trends are the same for both: the higher the sensitivity, the lower are the specificity, the P(active), and the EF. The behavior is the same for single structure and ensemble docking.

In the light of the docking results, we decided to select for the rest of the study the smina-dkoes scoring function and the single structure docking approach (using the 1QKU PDB structure) and to select two potential scoring TH (−6 and −7). Table 3 presents the performance of the selected protocols on the EPA database and the external validation sets (EADB and NR-DBIND) in terms of specificity, sensitivity, and binders retrieval rate.

### 2.3. Pharmacophore Modeling

#### 2.3.1. LB Pharmacophore Models

**Figure 3:**
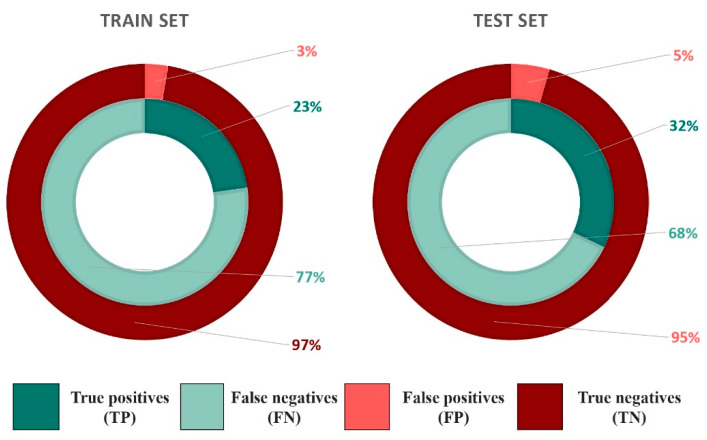
Pie charts displaying the performance of the combination of structure-based and ligand-based SBLB pharmacophores for the train and the test sets.

Since the compounds of the active training set belong to different chemical series, their alignment to derive a single LB pharmacophore is not feasible. To overcome this issue, all the compounds were clustered to obtain subsets of similar compounds for which pharmacophores can be generated. Distance between each cluster was fixed to 0.4 to ensure balanced groups and to minimize the number of singletons. In total, 14 clusters were obtained containing a minimum of 3 and a maximum of 69 compounds per cluster. 6 molecules could not be fitted in any cluster and were not used to generate the pharmacophores models. The maximum number of pharmacophores generated per cluster was set to 10. Each pharmacophore was used to screen the training subset of the EPA database. Based on individual hit retrieval performances, the best pharmacophore of each cluster was optimized according to the procedure described in the methods section. In the case where the optimization protocol failed, i.e., the optimized pharmacophore was not associated with a high rate of B/NB, the other pharmacophores generated for this cluster were considered in the descent order of their individual performances until one pharmacophore could be successfully optimized. If none out of the 10 generated pharmacophores or the corresponding optimized were associated with a high rate of B/NB, no pharmacophore was conserved for this cluster. In total, 11 unique (non-redundant) LB pharmacophores were obtained. Their performances in terms of selectivity and sensitivity are described in Table 4. These 11 LB pharmacophores were combined and used to screen the training subset of the EPA database. High specificity and relatively low sensitivity values were obtained with 30% of the total of binders retrieved against only 2.7% of the total of NB for the training set (Figure 3). To ensure that the performance is not biased towards the ligands of the training set, the 11 LB pharmacophore models were used to screen the test subset of the EPA database. Specificity and sensitivity values obtained were similar to those obtained with the training set and 27% of all B compounds were retrieved against 3% of all NB compounds (Figure 3).

#### 2.3.2. SB Pharmacophore Models

In addition to LB pharmacophores, 31 SB pharmacophores were generated from the holo structures of ERα available in the NR-DBIND. All these pharmacophores were used to screen the training set and were optimized according to the protocol described in the methods section. Redundant pharmacophores were removed, and 15 SB pharmacophores were retained. Screening of the EPA training and test subsets using the 15 SB pharmacophores led to low sensitivity values and high specificity values (Table 4). The percentage of B compounds retrieved with SB pharmacophores is similar to those obtained with the LB pharmacophores, but the percentage of NB compounds retrieved with the SB pharmacophore is lower.

#### 2.3.3. SBLB Pharmacophore Models

Results for both SB and LB selective pharmacophores were combined into a set of SBLB pharmacophores for ERα binding compounds. Redundant pharmacophores were removed to obtain a total of 26 unique SBLB pharmacophores. Performance in terms of sensitivity and specificity of this ensemble of pharmacophores is shown in Table 4. The set of SBLB pharmacophores is able to retrieve almost 40% of B against only 3% of NB.

The 26 SBLB pharmacophores were also used to screen the two external validation sets, i.e., the EADB and the NR-DBIND ERα sets, and the results are shown in Table 4. For EADB, similarly to the results associated with the EPA database, high specificity and low sensitivity values were obtained. The opposite is observed with the NR-DBIND ERα set, for which the sensitivity value is higher than the specificity.

### 2.4. Combination of Docking and Pharmacophore Models

Individual performances for docking (AUC, Se, and Sp) and pharmacophore models (Se, Sp, and hits retrieval rate) remain moderate, since sensitivities are hardly higher than 50% and the specificities equal or superior to 50% are associated with a low hit rate. For this reason, we evaluated the performance of the combination of docking and pharmacophore models in accurately predicting the binding profile of the compounds to ERα.

Two different protocols for performing this combination were explored, i.e., the consensus and the hierarchical protocols, detailed in the method section.

#### 2.4.1. Consensus Protocol

Using the consensus protocol, each molecule predicted as active using the docking or the pharmacophores models will be identified as an active compound in the consensus protocol results. The remaining compounds will be predicted as inactive. Performances obtained using this protocol for the EPA database and the validation datasets, i.e., the EADB and the NR-DBIND ERα set are depicted in Table 5.

Two docking TH defined using the PC were studied. For TH = −7, a sensitivity of 0.56 and a specificity of 0.76 are obtained for the EPA database. Conversely, for each validation set, the consensus protocol yields higher sensitivity (0.832 and 0.495, respectively) against lower specificities (0.495 and 0.029). When TH = −6 is chosen, the corresponding sensitivities are high: 0.81, 0.937, and 1 corresponding to the EPA database, the EADB, and the NR-DBIND, respectively. Recorded specificities are very low: 0.51, 0.158, and 0.005 for the EPA, the EADB, and the NR-DBIND. The higher positive predictive value (PPV) for the EPA database is reached by applying the TH = −7, with a PPV value around 19%. The same trend is observed with the EADB external validation set, whereas quite similar PPV are obtained for both threshold using the NR-DBIND set. The PPV obtained with the external validation sets using both TH = −6 and TH = −7 were largely superior to those obtained with the EPA database.

For equal specificity values between both TH, the TH = −6 yields better sensitivities for the EPA database as well as for the validation datasets. This is why our choice of docking TH is set at −6 for the consensus protocol.

#### 2.4.2. Hierarchical Protocol

We first evaluated the impact of using hierarchical screening with the pharmacophore models prior to or after the molecular docking models on the performance in enrichment.

Since both protocols displayed similar performances in terms of sensitivity and specificity, we relied on computational times to select the protocol. We thus decide to first screen using the pharmacophore models and then using the optimal docking protocol previously defined. On a desktop computer with 8x Intel(R) Xeon(R) CPU L5520 @ 2.27 GHz it takes ~75 min to dock the 2442 molecules against one ERα structure versus ~5 min to screen the same number of compounds on the 26 SBLB pharmacophore models.

Results depicted in Table 5 are those obtained using this hierarchical screening, i.e., the entire database is screened using the pharmacophore models and the compounds thereby identified as hits are used as the screening database for the docking method. The docking outcomes are then analyzed using the 2 docking scores TH previously identified and corresponding to different sensitivity values. For both TH values, the same trend is observed, i.e., high specificities (0.99 and 0.98) and low sensitivities (0.25 and 0.32).

Table 4 also presents sensitivity, specificity, and PPV obtained using the hierarchical protocol on the validation sets. The performance associated with the EADB is very similar to those obtained with the EPA database whereas the hierarchical protocol applied on the NR-DBIND ERα set lead to high values of sensitivity and specificity for both thresholds. Based on the hierarchical protocol outcomes, in particular the sensitivity values, on both the EPA database and the external validation sets, we selected the TH = −6 as the threshold to be used for docking scores using the hierarchical protocol.

## 3. Discussion

Through this work, we aim at finding the best in silico protocol(s) to discriminate B from NB compounds for ERα. Both SB and LB methods were evaluated, together with two different protocol to combine them.

### 3.1. Compounds and Database Preparation

The comparison of the distribution of the 15 constitutional descriptors for the three databases, i.e., EPA, EADB, and NR-DBIND, was performed in order to ensure that the difference in activity was not solely explained by the difference in physiochemical properties.

In order to assess the prediction performance of our models, we used external validation sets. Pairwise comparison of topological fingerprints between the EPA database and each external validation sets verifies the structural dissimilarity between those sets and thus the possible use of the EADB and NR-DBIND ERα sets as external validation sets. Moreover, the SALI map confirms that the three databases belong to the same chemical space, which was recommended for pharmacophore models validation [27].

### 3.2. Docking

For the docking approach, both single structure and ensemble docking were explored. Three software with free academic licenses, accounting for 6 scoring functions, were used, i.e., smina (smina-ad4, smina-dkoes, smina-vina, smina-vinardo), Surflex-dock, and PLANTS. Although different magnitudes of AUC were obtained, most of them agreed on the elected structure yielding the best single structure docking results: 4 out of the 6 docking methods associated the best outcomes with the 1a52 structure. However, the highest AUC were obtained with different structures, 1qku and 1x7e for smina-dkoes and PLANTS, respectively. Interestingly, the 1a52 structure presents an artifactual position of the helix that is extending away from the body of the ligand binding domain. The resulting conformation is more similar to an antagonist-bound ERα structure than an agonist-bound one [4]. This observation leads to discard 1a52 despite its selection by most of the software and reinforces the choice of the 1qku structure and smina-dkoes as the optimal single structure docking protocol. It is to note that 1qku is co-crystallized with the native ligand 17β-estradiol. Furthermore, it was shown that smina_dkoes was very proficient at sampling low RMSD poses compared to Vina [28].

No major performance improvement as evaluated by the AUC values was brought by ensemble docking over the single structure strategy. This was true using either only agonist-bound structures ensembles or combinations of agonist and antagonist-bound structures. When considering only agonist compounds as positives and the remaining compounds (antagonists and experimental non binders) as negatives, both agonist-bound and antagonist-bound structures were associated with similar AUC values (results not shown). Similarly, no significant differences in docking performance were noted among agonist- and antagonist-bound structures when only antagonists were set as positives and all the remaining compounds as negatives. This could be explained by the fact that ERα conformations used in this study are very similar, as shown by the RMSD values obtained among all structures (Table S4). The structures used display a limited flexibility, explaining the similar performances obtained in terms of AUC values, regardless of the pharmacological profile of the co-crystallized ligands or of the binding compounds. This limited flexibility sampling can also explain the lack of significant performances improvement observed using the ensemble docking strategy. Furthermore, previous studies also showed that ensemble docking did not always outperform the single structure docking approach especially when the single structure is rationally selected [29]. Finally, and although displaying several advantages, such as accounting for the flexibility of the target, ensemble docking presents also noteworthy drawbacks. Docking a database against more than one protein structure requires more computational resources and/or time. Ensemble docking can also lead to inaccurate predictions due to a favored inaccurate interaction with a particular protein conformation included in the ensemble [30].

### 3.3. Predictiveness Curve

**Figure 4:**
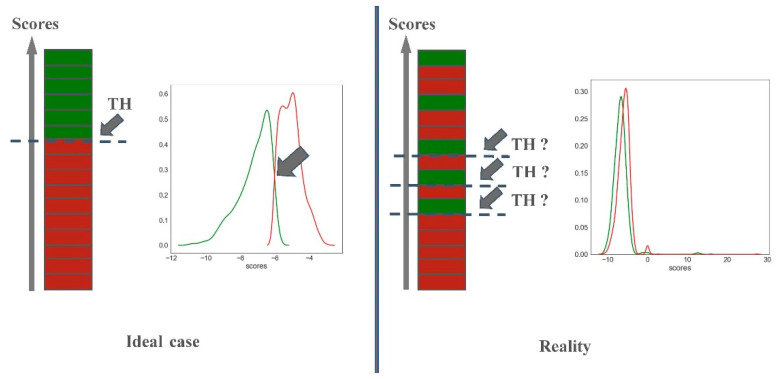
Docking scores distribution between B (green) and NB compounds (red) of the EPA database.

Molecular docking is a valuable method often used to elucidate a mechanism of action or to predict the nature of interactions established between a ligand and a target protein. It can also be used as a screening tool to filter a database according to docking scores. In a virtual screening protocol using molecular docking, the ranked list of compounds according to the docking scores is generated. Then, a fraction of the top scoring compounds (1%, 5%, 10%…) is tested experimentally depending on the budget and experimental facilities. For this type of protocol, defining a docking score threshold is not necessarily a priority. In our study, we preferred to rationally select an optimal docking threshold rather than selecting an arbitrary fraction of the top scoring compounds. Endocrine disruptome [21], for example, is an online tool based on docking calculations that also established docking scores thresholds to differentiate between binding and non-binding compounds for a set of NRs. In an ideal case where all B compounds would have better docking scores than the NB compounds (Figure 4, left panel), the threshold would simply be defined as the value separating the docking score values of the last ranked B compound and the first ranked NB compound. However, in reality, some B and NB compounds present very similar docking score values and the distribution of the profiles of scores between B and NB compounds are often overlapping. In our study, both distribution curves for B (green) and NB compounds (red) overlap (Figure 4, right panel), preventing a straightforward manual definition of a perfect score threshold. To help the definition of a score threshold, we used Screening Explorer [31], an interactive tool for the analysis of screening results, based on the predictiveness curve (PC) metric [32].

Although newly introduced in the virtual screening field, the PC has already been applied in different studies [27,33,34,35,36,37]. This metric is usually used altogether with ROC curves and enrichment factors to assess the ability of a given method to discriminate active compounds from inactive ones [38]. PC have been used in the literature to define a score threshold to discriminate agonist from antagonist compounds for androgen receptors [27]. As in [27], we assessed the predictiveness of the single structure and ensemble docking approaches as well as each docking/scoring scheme. Using the Screening Explorer tool, 2 potential docking score thresholds were identified to differentiate ERα B from NB. We thus chose to evaluate these 2 docking score thresholds for the combination of the docking procedure and the pharmacophores modeling.

### 3.4. Pharmacophores

**Figure 5:**
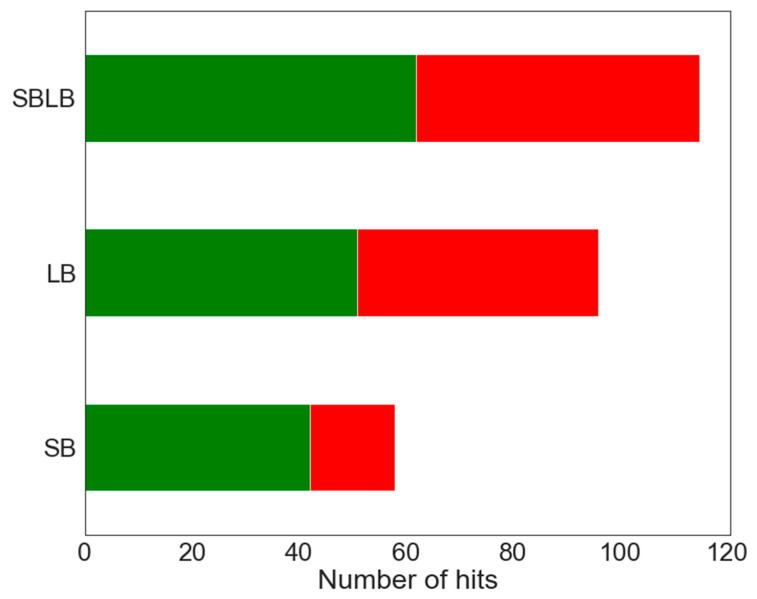
Barplot displaying the proportion of B (green) and NB (red) retrieved as hits using SB and LB pharmacophore models individually and with the SBLB combination.

Several studies already focused on generating pharmacophores for NRs ligands [27,39,40,41,42]. In this work, numerous SB and LB pharmacophores targeting ERα were generated and optimized. A large number of B were retrieved by both SB and LB pharmacophores, but some were specifically identified by only one or the other class of pharmacophores. Consequently, all non-redundant pharmacophores were merged in the SBLB ensemble that contains approximately as much LB (11) as SB (15) pharmacophores. The SBLB ensemble of pharmacophores achieve better sensitivity over a slight drop in the specificity compared to SB pharmacophores or LB pharmacophores. Hits retrieved by the SB and LB pharmacophores are represented in Figure 5 together with the yield of the SBLB pharmacophores.

Interestingly, our SBLB pharmacophores applied to the external validation data yielded very good sensitivities and lower specificities. This is similar to the results obtained by Réau et al. [27] with pharmacophores models generated using the NR-DBIND AR set. This study also suggests that pharmacophores are only suited for data filtering as long as the compounds belong to the same chemical space as the molecules used to build the model. The SALI map of all the databases (the EPA training database and the EADB and NR-DBIND ERα external validation sets) in Figure 2 shows that our data fit this requirement and supports the use of pharmacophores for this study. The lower sensitivities obtained with the EPA database compared to those obtained with the external validation sets may be explained by the imbalance in the number of B and NB that exists in the EPA database (223 B and 2219 NB) compared to the validation sets which present lower proportions of inactive data. The SBLB pharmacophores present better performance in discarding true negatives than in identifying true positives. To overcome this issue, we decided to evaluate the ERα B prediction performances obtained when combining SBLB pharmacophores and docking approaches.

### 3.5. Combination of Methods

**Figure 6:**
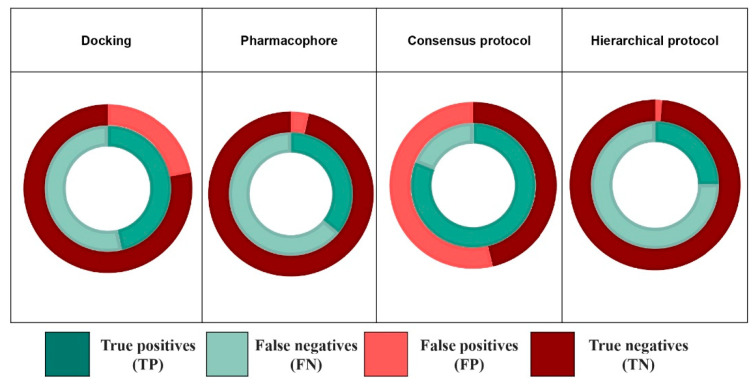
Pie charts illustrating the performance of each individual model (docking and pharmacophores) and the combination of both using the consensus and the hierarchical protocol.

Combining several bioinformatic methods is often used for various purposes such as extending the knowledge about a drug–target interaction or refining screening results [43,44,45,46,47]. Docking methods are usually successful in poses prediction but fail at distinguishing active from inactive compounds yielding low sensitivities. Pharmacophore methods on the other hand, used in the appropriate applicability domain [27], succeed at discarding molecules which structures misfit the requirements to interact with the binding site. In accordance with these results, our study shows that the 2 types of combinations we evaluated enhance performances towards better specificities for the hierarchical protocol and better sensitivities for the consensus protocol (Figure 6).

Furthermore, a review of studies dedicated to NR, and more specifically to the prediction of EDCs able to bind ERα, enabled us to better assess the performances obtained with our models. We obtained high sensitivities values, 0.81 for EPA and 0.93 and 1 for EADB and NR-DBIND, respectively, associated with low specificities. The different studies herein undermentioned can be divided into studies relying on docking models and others that are mostly based on machine learning and QSAR (Quantitative structure activity relation) models [20,23,24,48,49,50,51,52,53]. Docking methods of the studies of the former class [21,22,54,55,56,57] present AUC values similar to those obtained with our selected scoring function and receptor structure. It should be noted that these docking studies used various ERα structures, and especially the 1a52 we chose to discard because of its artifactual position of the helix 12 [4]. Studies of the latter category are the most abundant, and present high AUC values around 0.8 with good overall sensitivities and specificities. These good prediction performances are not surprising since classification and QSAR models are known for their ability to well predict structural analogs. However, these methods can suffer from overfitting bias which can lead to lower performances if applied on a different dataset as they will be unable to predict completely new/different molecules [58]. Moreover, outliers are frequently discarded in this kind of study, but these compounds may introduce a category of yet unrepresented compounds. Nevertheless, these LB methods perform better than our LB pharmacophores and should be investigated for future integration in the protocol.

Some sources of bias that may have affected the performances should be taken into consideration. Annotation errors of biological assays are possible, and compounds identified with binding assays may bind on different ERα binding sites. Furthermore, the compounds of the EPA database are mostly compounds suspected to be toxic and not therapeutic compounds. Even if our models were validated with external sets dedicated to therapeutic compounds, it is important to enrich databases with more compounds relative to both therapeutic and toxicological explorations according to the purpose of the study [59,60].

Previous studies [27,49,55,61] suggested that the pharmacological profile of the ligands should be considered to better discriminate agonist from antagonist compounds. Endocrine disrupting chemicals act in several ways including agonism and antagonism [17] and it is important to be able to retrieve ERα B regardless of their pharmacological profiles. The structure identified to be optimal for the docking study is in an agonist-bound conformation. We thus verified that our protocol was not biased towards agonist ligands and that we were also able to identify ERα B with different pharmacological profiles.

**Figure 7:**
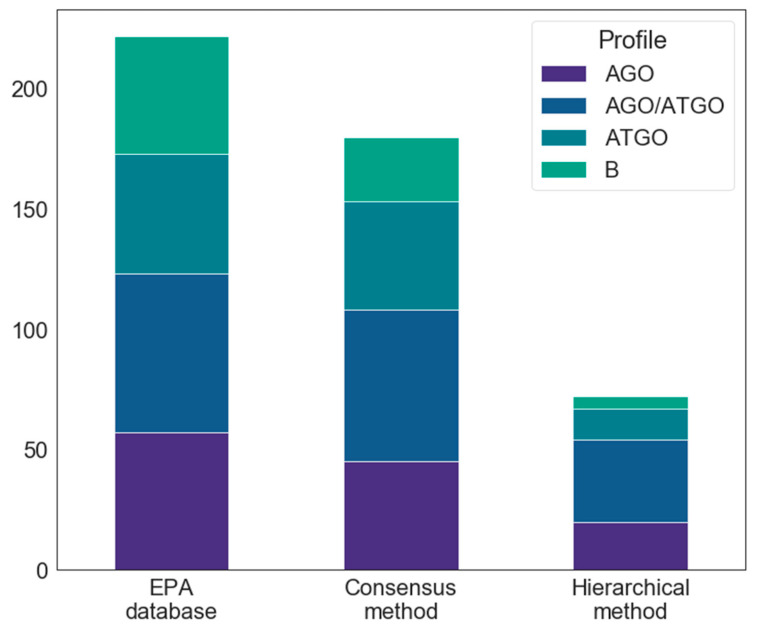
Barplots illustrating the relative proportion of compounds of each pharmacological profile (AGO: compounds with ERα agonist activity, ATGO: compounds with ERα antagonist activity, AGO/ATGO: compounds with both ERα agonist and antagonist activities, B: ERα binders without pharmacological profile annotation) in the EPA database and among the hits identified using the hierarchical and consensus protocols.

We compared the distribution of pharmacological profiles within the starting database, i.e., the EPA database, as well as within the hits obtained for each screening protocol (see Figure 7). In the EPA database, the pharmacological profile annotation was achieved using agonist and antagonist experimental assays. Among the 223 compounds, 58 are agonist (26%), 50 antagonist (22.4%), and 66 agonist–antagonist (29.6%) compounds. No pharmacological profile annotation was available for 49 molecules (22%). Interestingly, the relative proportion of each pharmacological profile observed in the initial EPA database was maintained among the hits of both consensus and hierarchical protocols. This highlights the fact that the screening protocol presented herein is able to identify ERα B, regardless of their pharmacological profile and is thus not overfitted towards any pharmacological profile.

Finally, it is important to mention that both sensitivity and specificity are valuable for assessing the screening quality. However, and depending on the purpose of the study, one value tends to be more meaningful than the other. Therapeutic studies favor good specificities as they are an indicator of the ability to discard true negatives, which is more important to reduce the number of molecules to be tested in vivo. For toxicological studies, high sensitivities are preferred, as the goal is to identify the maximum of potentially undesired compounds. These observations are supported by the results obtained for validation sets. In this way, we suggest that the consensus protocol is better tailored for our study and the hierarchical protocol could better suit drug design projects. Both protocols provide a list of compounds that are predicted to bind ERα. These predictions must be confirmed and the estrogenic activity modulation and potential endocrine disruption effects should be further experimentally assessed.

## 4. Materials and Methods

### 4.1. Compounds, Databases Preparation, and Annotation

Two types of dataset were used, i.e., a set formed by EPA compounds used to build the different individual methods and two external data sets (the NR-DBIND, the EADB database) used for validation.

#### 4.1.1. EPA Dataset

Compounds and biological data used to build the training dataset were extracted from the United States Environmental Protection Agency (EPA). Chemical compounds and their associated biological data were downloaded from the DSSTox dashboard in February 2019. The platform has been removed since then and compounds can now be found under the Comptox dashboard [62]. This dashboard gathers high throughput screening data of a large and structurally diverse chemical library of compounds sus-pected to be of risk for humankind and for the environment against a wide spectrum of biological targets involved in toxicity pathways [63]. Compounds included in training dataset were obtained by filtering the DSSTox/Comptox database to only keep compounds that have undergone binding assays on ERα receptor. All compounds were available in csv files where each molecule was identified by its SMILES and CAS number. Binding compounds were selected to form the active subset (activity annotated 1) and the non-binding molecules constituted the inactive subset (activity annotated 0). This data-base will be referred to as the “EPA database”. The EPA database is available in the Sup-plementary Materials in SMILES format.

#### 4.1.2. Validation Sets

- NR-DBIND

The NR-DBIND (Nuclear Receptors DataBase Including Negative Data) is a non-commercial manually curated benchmarking database that provides affinity data for small molecules that were experimentally tested against 28 nuclear receptors [64]. For this study, a filter was applied to extract compounds tested against ERα. All compounds were directly downloaded from the website (http://nr-dbind.drugdesign.fr/, accessed on 20 November 2019) in SMILES format and annotated by their CAS names. Binding compounds were selected to form the active subset and the non-binding molecules constituted the inactive subset.

- EADB

The Estrogenic Activity Database (EADB) developed by the NCTR (National center for toxicological research) assembles a large number of estrogenic activities data from various sources [56,65,66]. It contains 18.114 estrogenic-activity data points collected for 8212 chemicals tested in 1284 binding assays, reporter-gene assays, cell-proliferation assays, and in vivo assays in 11 different species. The database has been directly downloaded from the website (https://www.fda.gov/science-research/bioinformatics-tools/estrogenic-activity-database-eadb, accessed on 25 November 2019) and filtered to only keep data relative to human ERα.

#### 4.1.3. Molecule Curation and Preparation

The same molecule curation and preparation protocol was applied for the EPA database, the NR-DBIND, and the EADB validation sets. SMILES were standardized using Standardizer from the ChemAxon suite [67] and salts and fragments were removed together with duplicates and small molecules containing less than 5 atoms. Conformations were generated using i-Con [16], the conformer generation tool of LigandScout [68], with BEST settings except for the maximum number of conformations per molecule that was set to 25. Compounds containing certain metal atoms (e.g., Pb or Hg) were removed from the docking collection mainly because the software used were unable to process these molecules. Finally, molecules were converted into the appropriate format for the different software at use, i.e., pdbqt for docking with smina, mol2 for PLANTS, and Surflex_dock and ldb for pharmacophore model generations.

In order to assess the accuracy of the data, 15 constitutional, physiochemical, and molecular descriptors were computed for each molecule of the three databases, namely, molecular weight (MW), ClogP, ClogS, number of HBond-Acceptors (H-Acc), number of HBond-Donors (H-Don), Total Surface Area (TSA), Relative Polar Surface Area (RPSA), Shape Index, Molecular flexibility (Mol_Flex), Molecular Complexity (Mol_Comp), number of Electronegative atoms (Elect_atom), number of Stereo Centers (Stereo_cent), number of rotatable bonds (rotat_bond), number of aromatic rings (aromatic_rings), and number of aromatic atoms (aromatic_atom). Descriptors were computed using the DataWarrior software [26]. Moreover, topological fingerprints were computed using the rdkit library [69] for python and pairwise Tanimoto coefficient (Tc) were calculated between compounds of the EPA database and the EADB on one side and EPA database and the NR-DBIND on the other side.

### 4.2. Structures Preparation

ERα structures were selected according to 3 criteria: (1) human structures; (2) without mutations nor residue’s deletion in the ligand binding domain; (3) referenced by a scientific article. Accordingly, 31 holo structures were used for SB pharmacophore building. Among these 31 structures, only 7 holo (Protein-ligand) crystal structures of human ERα were used for docking (Table S5). The 24 remaining structures were discarded since they presented residues deletion in the binding site that can affect docking results more than pharmacophore building. Among these 7 structures, 2 are classified as antagonist bound as they are co-crystallized with an antagonist molecule. The remaining 5 structures are agonist-bound and 4 of them share the same co-crystallized ligand, the 17β-estradiol. For the docking procedure, the structures were directly downloaded from the NR-DBIND database [64] since they are already enumerated, annotated, and cleaned. Format conversion from PDB to the appropriate docking format was done accordingly to the requirements of the software, i.e., PDB were converted into mol2 format using the software chimera [70], into pdbqt with the prepare_receptor4.py python script available with the MGLTool [18]. In order to generate the structure-based pharmacophores, structures were directly downloaded from the RCSB website [71] via the LigandScout graphical interface.

### 4.3. Docking

#### 4.3.1. Protocol

Docking is a structure-based virtual screening method that aims at predicting the pose of a ligand inside a protein [17]. All docking calculations were performed with 3 different software with free academic licenses, i.e., smina [72], PLANTS [73], and Surflex-dock [74]. The same binding site was used with the 3 software that was delimited using the co-crystallized ligands. For each software, 5 docking runs were performed.

Smina is a fork of AutoDock Vina [28] that is designed for scoring function development and minimization workflows [72]. It relies on the same sampling algorithm as vina, the latter being the succession of stochastic mutations steps, but integrates several scoring functions. For this study, we relied on 4 scoring functions already implemented within smina, i.e., vina [28], the Vina RaDii Optimized (vinardo) [75], dkoes [72], and ad4 scoring functions. All dockings were performed using the default options of smina and num_modes = 20 and exhaustiveness of 8. The bounding box coordinates were determined based on the crystal structure of 1a52 used as reference to align the remaining structures. The box parameters were chosen based on the co-crystallized ligand position with a spacing of 1 Angstrom. A cubic box was delimited with size_x, size_y, and size_z set to 20 and the following coordinates center_x = 107.175, center_y = 14.983, and center_z = 96.009. PLANTS relies on the docking algorithm carrying the same name. This Protein–Ligand ANT System (PLANTS) algorithm is based on ant colony optimization, a class of stochastic optimization. An artificial ant colony must find the minimum energy conformation of the ligand within the receptor through a trail of pheromone whenever an ideal low energy conformation is found. This marking is iteratively changed until the lowest energy conformation is found [73,76,77]. The binding site coordinates were the same that were used for smina. Regarding other parameters, the binding site_radis was set to 18, the cluster_structures to 10, the cluster_RMSD to 2, and the search speed to “speed2”.

Surflex-dock is a docking methodology that combines Hammerhead’s empirical scoring function with a molecular similarity method to generate putative poses of ligand fragments [74]. The search approach is based on an incremental construction and a fragment assembly method similar to the genetic algorithm. Surflex-Dock uses a pseudo-molecule, a protomol, as a target to align fragments of the ligands. Protomols were generated starting from the holo structures.

#### 4.3.2. Docking Performances Analyses

- Single structure docking and ensemble docking

In the single structure docking approach, docking performance for each PDB structure was evaluated individually by calculating the area under the ROC curve (AUC). AUC values were computed with python using the *scikitlearn* library [78] and the package *sklearn metrics*. In the ensemble docking approach, docking performances of all the possible ensembles of 2, 3, 4, 5, 6, and 7 structures were computed. In this approach, each ligand was sequentially docked into several protein structures. The results were post processed to keep only, for each ligand, the best docking score among all structures. All ligands are then ranked according to these new scores and the corresponding AUC are computed. Python version 3.8.1 was used to prepare data and analyze the results.

- Predictiveness curves

Although docking scores are continuous values, they can be transformed into a binary classifier to discriminate between ERα B and NB using the predictiveness curve (PC) [27,32]. The predictiveness curve is a metric usually used in clinical epidemiology to evaluate the ability of a biological marker to assess the fit of risk models and to estimate the clinical utility of a model when applied to a population [32]. Transferred to the field of Chemoinformatics, this metric can be used to assess the predictive power of a screening methods as well as defining a score threshold retrieving best candidates to be tested experimentally. In this way, PC was used to define a docking scoring threshold for which we can compute the probability that a compound with this given score will be a B compound and define associated sensitivity (Se) and specificity (Sp) (*cf* Equations (1) and (2)). Enrichment factor $EF_{x\%}$ and positive predictive value (PPV) were also calculated following the Equations (3) and (4) where $Hits_{x\%}$ is the number of active compounds in the top x% of the ranked dataset, $Hits_{t}$ is the total of active compounds, $N_{x\%}$ is the number of compounds contained in the x% of the dataset, and $N_{t}$ is the total number of compounds in the dataset.

(1) $$Sensitivty=\frac{Nb\;of\;True\;Positives\;}{Nb\;of\;True\;Positives+Nb\;of\;False\;Negatives}$$

(2) $$Specificity=\frac{Nb\;of\;True\;Negatives}{Nb\;of\;True\;Negatives+Nb\;of\;False\;Positives}$$

(3) EFx%=Hitsx%Nx%HitstNt

(4) $$PPV=\left[\frac{Nb\;of\;True\;Positives}{Nb\;of\;True\;Positives+Nb\;of\;False\;Positives}\right]\times 100$$

The aim of the study is to select as much positive data as possible (toxic compounds). It is then interesting to identify a TH associated with a high probability of activity P(active) but also a high value of sensitivity (Se).

Various TH values and their P(active), Sp, PPV, and EF were calculated for different sensitivity values (0.25/0.5/0.75). The highest P(actives)max was calculated beforehand for each scoring function and for the different ensemble sizes.

### 4.4. Pharmacophore Modeling Protocol

Structure based (SB) and ligand based (LB) pharmacophores were generated using LigandScout software version 4.4 [68].

#### 4.4.1. Ligand Based Approach (LB) Models Protocol

In order to generate LB-pharmacophores, active compounds from one side and inactive compounds on the other were both divided into training and test sets. 75% of the active compounds and 75% of the inactive compounds were gathered to form the training set. The remaining 25% of active compounds and inactive compounds were used to form the test set. The active compounds of the training set were clustered using the i-cluster [79] tool provided with LigandScout software and pharmacophores were generated for each of the resulting clusters. Default parameters of the I-cluster tool were used, i.e., cluster_dis = 0.4 with average method, except for the maximum number of conformations set to 3. In order to derive a LB-pharmacophore dedicated to a particular cluster of compounds, LigandScout operates in several steps: (1) conformations of the ERα ligands included in the cluster are generated using the ICON algorithm; (2) molecules are ranked according to their flexibility and the best alignments; (3) for each compound, the generated conformations are used to create intermediate pharmacophores that are ranked using several scoring functions; (4) common features are aligned to all the conformations of the next molecule and so on until all the molecules are processed [80]. Each final pharmacophore obtained with this protocol was used to screen the train set on which global and individual performances were assessed. In order to make sure that data separation into training and test does not affect the performance, the whole procedure (from training and test set separation to pharmacophores generation and evaluation) was repeated 25 times. The iteration yielding the best global performances was kept and used during the pharmacophore optimization set and the composition of each set.

#### 4.4.2. Structure Based Approach (SB) Models Protocol

3D SB pharmacophores were automatically generated from the PDB structures of ERα included in the NR-DBIND [64]. In this approach, the LigandScout algorithm tags the key features of the ligands that are interacting with the residues of the receptor. To complete the pharmacophore, an ensemble of exclusion volume spheres is generated to represent the shape of the active site [42].

- Pharmacophore model optimization

In order to optimize the pharmacophore, we followed literature recommendations, especially a screening protocol that succeeded in generating selective pharmacophores for NR agonist ligands and selective pharmacophores for NR antagonist ligands [42]. This protocol was applied on both SB and LB pharmacophores. The generated 3D pharmacophores were used to screen the training set and the test set. All the ligands were converted into ldb format using the idbgen tool provided with LigandScout. For each pharmacophore, a first screening was made with LigandScout default settings and particularly the Max. number of omitted features set to 0. Two case scenarios were possible. If after the first screening, the ratio PPV was high, i.e., few non binders are retrieved but a large number of binders are matching the pharmacophore, a second screening was performed with the same pharmacophore but setting the Max. number of omitted features to 1. This way, non-essential features could be identified to be removed or set as optional possibly leading to the retrieval of more active compounds and less inactive molecules. After that, a third screening was performed with Max. number of omitted features set to 0 again. If the ratio of PPV decreased, this pharmacophore was not validated, and another round of feature identification was performed. If the ratio increased, the pharmacophore was validated, and other potential non-essential features were investigated. This protocol was applied to each pharmacophore until 3 pharmacophoric features were retained or until no non-essential features could be identified.

#### 4.4.3. Combination of SB and LB Pharmacophores Models

Once a collection of optimal SB and LB pharmacophores was obtained, redundant pharmacophores were removed. Redundant pharmacophores are pharmacophores that can be removed without decreasing the recall, i.e., pharmacophores that only retrieved ligands that are also retrieved with other pharmacophores of the set. To remove these redundant pharmacophores, all generated pharmacophores were ranked according to the number of hits they retrieved. Then, each pharmacophore was removed sequentially, starting from the pharmacophore associated with the smallest number of hits. For each removal, the impact on the recall was evaluated. If the recall was not affected, the pharmacophore was dismissed and, in the opposite, if the recall decreased, the pharmacophore was conserved.

The SBLB pharmacophores used in this study are available in the Supplementary Materials in pml format.

### 4.5. Pipelines Construction

Two different ways of combining pharmacophore models and docking were explored, the consensus and the hierarchical protocols. The first protocol consists in the analysis of the union of the results belonging to each model. Each molecule predicted as active by docking or pharmacophore will be predicted as active compound by the consensus protocol. The remaining compounds will be predicted as inactive. The second approach used a hierarchical protocol in which the database undergoes a sequence of screening methods. Two possible sequences exist: [pharmacophore-docking] or [docking-pharmacophore].

## 5. Conclusions

In the present work, we present a pipeline designed for the prediction of potential EDCs acting through the binding to ERα. Optimized protocols for docking studies and SB and LB pharmacophore models’ generation were evaluated together with the best approach to combine them. Both combination approaches that were investigated here, i.e., consensus protocol and the hierarchical protocol, yielded good results. However, we recommend favoring the consensus protocol for toxicological studies and the hierarchical protocol for the identification of therapeutic compounds. Results were validated using two external datasets. Using our pipeline, we show that combining several in silico methods can enhance the prediction performances for compounds binding to ERα. Additional methods should be evaluated and implemented in this pipeline such as classification models.

## Figures and Tables

**Table 1:** Docking performances (Max area under the ROC curve (AUC), min, mean, and standard deviation (SD)) calculated for the different scoring functions and for the different docking approaches.

Software	Docking Approach	Best Performances		Min AUC	Mean AUC	SD
		AUC	PDB			
smina-dkoes	Single	0.708	[1qku]	0.700	0.704	0.003
	Ensemble of 2	0.709	[2yja-1qku]	0.702	0.703	0.003
	Ensemble of 3	0.710	[2yja-1qku-1g50]	0.704	0.702	0.003
smina-vina	Single structure	0.699	[1a52]	0.643	0.676	0.02
	Ensemble of 2	0.696	[1xp9-1a52]	0.642	0.67	0.017
	Ensemble of 3	0.695	[1xp9-1xp1-1a52]	0.642	0.667	0.014
smina-vinardo	Single structure	0.68	[1a52]	0.686	0.704	0.018
	Ensemble of 2	0.676	[1xp9-1a52]	0.619	0.650	0.019
	Ensemble of 3	0.673	[1xp9-1xp1-1a52]	0.618	0.644	0.018
smina-ad4	Single structure	0.656	[1a52]	0.613	0.639	0.0154
	Ensemble of 2	0.654	[1x7e-1a52]	0.618	0.641	0.009
	Ensemble of 3	0.650	[1x7e-1qku-1a52]	0.623	0.640	0.007
PLANTS	Single structure	0.659	[1x7e]	0.598	0.634	0.019
	Ensemble of 2	0.660	[1x7e-1a52]	0.647	0.62	0
	Ensemble of 3	0.659	[1x7e-1qku-1a52]	0.620	0.642	0.009
Surflex-dock	Single structure	0.604	[1a52]	0.547	0.576	0.027
	Ensemble of 2	0.616	[1xp1-1x7e]	0.556	0.594	0.020
	Ensemble of 3	0.623	[1xp1-1x7e-1a52]	0.562	0.605	0.015

**Table 2:** P(active), scoring threshold (TH), Specificity (Sp), Enrichment factor (EF), and the positive predictive value (PPV) calculated for different values of sensitivity (Se) (0.25/0.5 and 0.75) for all the docking approaches and for the scoring function smina-dkoes and Protein–Ligand ANT System (PLANTS).

	Docking Approach	Performances	Se = 0.25	Se = 0.5	Se = 0.75
smina_dkoes	Single	P(active)	0.137	0.094	0.094
	(1qku)	TH	−7	−6	−6
		Sp	0.918	0.766	0.601
		EF	1.9	1.65	1.65
		PPV	56/237	111/631	167/1052
	Ensemble de 2	P(active)	0.134	0.094	0.094
	(2yja-1qku)	TH	−7	−6	−6
		Sp	0.916	0.759	0.597
		EF	1.89	1.63	1.63
		PPV	56/242	111/645	167/1061
	Ensemble de 3	P(active)	0.137	0.13	0.091
	(2yja-1qku-1g50)	TH	−8	−7	−6
		Sp	0.915	0.777	0.599
		EF	2.37	1.9	1.59
		PPV	56/244	111/605	167/1057
PLANTS	Single	P(active)	0.127	0.103	0.081
	(1x7e)	TH	−79	−72	−64
		Sp	0.876	0.723	0.501
		EF	1.9	1.69	1.42
		PPV	55/328	110/719	165/1261
	Ensemble of 2	P(active)	0.123	0.097	0.08
	(1x7e-1a52)	TH	−82	−73	−66
		Sp	0.86	0.707	0.49
		EF	1.69	1.58	1.42
		PPV	55/362	110/753	165/1287
	Ensemble of 3	P(active)	0.122	0.096	0.079
	(1x7e-1a52-1qku)	TH	−82	−73	−66
		Sp	0.857	0.701	0.493
		EF	1.65	1.6	1.41
		PPV	55/369	110/767	165/1279

**Table 3:** Sensitivities (Se), specificities (Sp), and positive predictive value (PPV) calculated for the single docking approach with smina_dkoes scoring function screening for both TH = −6 and TH = −7 scoring thresholds.

Scoring Threshold (TH)	Performances	EPA	Estrogenic Activity DataBase (EADB)	Nuclear Receptors DataBase Including Negative Data (NR-DBIND)
**TH = −7**	Se	0.79	0.48	0.93
	Sp	0.55	0.58	0.03
	PPV	176/2442	63/232	513/732
**TH = −6**	Se	0.46	0.77	0.99
	Sp	0.78	0.198	0.001
	PPV	103/2442	101/232	553/732

**Table 4:** Sensitivity (Se) and specificity (Sp) of ligand-based (LB), structure-based (SB), and combination LB and SB pharmacophores, for the training set and the test set of the EPA database, the EADB and the NRDBIND.

		EPA Database		EADB	NR-DBIND
	Performances	Train Set	Test Set	Validation Set	Validation Set
LB pharmacophores	Se (B/total_B)	0.305 (51/167)	0.232 (13/56)		
	Sp (NB/total_NB)	0.973 (45/1664)	0.960 (22/555)		
SB pharmacophores	Se (B/total_B)	0.251 (42/167)	0.232 (13/56)		
	Sp (NB/total_NB)	0.990 (16/1664)	0.987 (7/555)		
SBLB pharmacophores	Se (B/total_B)	0.371 (62/1664)	0.321 (18/56)	0.557 (73/131)	0.819 (458/554)
	Sp (NB/total_NB)	0.968 (53/167)	0.595 (25/555)	0.871 (13/101)	0.629 (66/178)

**Table 5:** Sensitivities (Se), specificities (Sp), and B/Total ratio calculated for the consensus and hierarchical screening method for two different thresholds (TH) of docking scores.

	TH	−7			−6		
		Se	Sp	PPV	Se	Sp	PPV
**Consensus protocol**	EPA database	0.56	0.76	124/652	0.81	0.54	180/1205
	EADB	0.832	0.495	109/160	0.931	0.158	122/207
	NR-DBIND	0.986	0.029	546/719	1.0	0.005	554/731
**Hierarchical protocol**	EPA database	0.25	0.99	55/84	0.32	0.98	72/117
	EADB	0.206	0.960	27/31	0.370	0.911	52/61
	NR-DBIND	0.756	0.635	419/484	0.814	0.635	451/516

## Data Availability

The reported data are provided in the Supplementary Materials.

## Supplementary Materials

Supplementary Materials can be found at https://www.mdpi.com/1422-0067/22/6/2846/s1, Figures S1 and S2: Boxplot of the distribution of the 15 physiochemical properties computed with Datawarrior for EADB (S1) and NR-DBIND (S2), Figure S3: Boxplot representing the distribution of pairwise calculated Tanimoto coefficient between EPA database and EADB (in blue) and NR-DBIND (green) topological fingerprints, Table S1: Docking performances for both single and ensemble docking approach illustrated with the AUC of the best and the worst ensembles; Table S2: Maximum values of predictiveness (P(active)) associated to each scoring function and each docking approach; Table S3: Sensitivity (Se), specificity (Sp), scoring threshold (TH), Enrichment factor (EF) and PPV calculated for the best scoring function (Smina-dkoes and PLANTS) and corresponding to P(active)max for different docking; Table S4: Pairwise RMSD computed between all the protein structures; Table S5: PDB Structures used for structure based model building. All 31 structures were used to generate SB pharmacophores and only those colored in blue were used for docking; EPA database in SMILES format; SBLB pharmacophores in pml format.

## Abbreviations

AR	Androgen receptors
AUC	Area under the ROC curve
B	Binding compounds
CAS	Chemical Abstracts Service
DBD	DNA-binding domain
DNA	deoxyribonucleic acid
DSSTox	Distributed Structure-Searchable Toxicity
EADB	Estrogenic activity database
EDCs	Endocrine disrupting chemicals
EF	Enrichment factor
EPA	United states Environmental protection agency
ER	Estrogen receptors
FIX	Factor IX
GR	Glucocorticoid receptors
LB	Ligand based
LBD	Ligand binding domain
NB	Non-Binding compounds
NCTR	National center for toxicological research USA
NR	Nuclear receptor
NR-DBIND	Nuclear Receptors Database Including Negative Data
NTD	NH2-terminal domain
PC	Predictiveness curve
PDB	Protein data bank
PPAR	Peroxisome proliferator-activated receptors
PPV	Positive Predictive value
PLANTS	Protein-ligand ANTSystem
QSAR	Quantitative structure activity relationship
RMSD	Root-mean-square deviation
ROC	Receiver operating curve
SB	Structure based
SD	Standard deviation
Se	Sensitivity
SMILES	Simplified molecular-input line-entry system
Sp	Specificity
TH	scoring Threshold
TR	Thyroid hormones receptors
