# Supplementary material for: Predicting Potential Endocrine Disrupting Chemicals Binding to Estrogen Receptor α (ERα) Using a Pipeline Combining Structure-Based and Ligand-Based in Silico Methods
Source: Int J Mol Sci. 2021 Mar 11;22(6):2846. doi: 10.3390/ijms22062846 (PMC7999354; doi:10.3390/ijms22062846)
Supplement: Supplementary file 1 [file ijms-22-02846-s001.zip › Sellami_et_al_Supp_info.docx]

Article

Predicting potential EDCs binding to ERα using a pipeline combining SB and LB in silico methods

Asma SELLAMI ^1^, Matthieu MONTES ^1+*^ and Nathalie LAGARDE ^1+*^

^1^ Laboratoire GBCM, EA 7528, Conservatoire National des Arts et Métiers, Hésam Université, 2 rue Conté, F-75003 Paris, France;

^+^ These authors contributed equally to this work

***** Correspondence: [nathalie.lagarde@lecnam.net](mailto:Nathalie.lagarde@lecnam.net) ; [matthieu.montes@cnam.fr](mailto:matthieu.montes@cnam.fr)


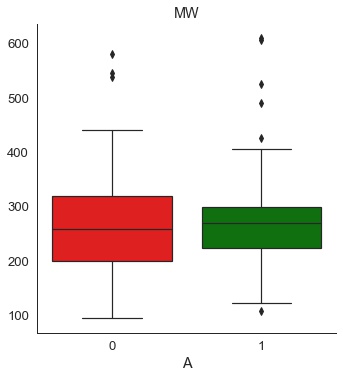

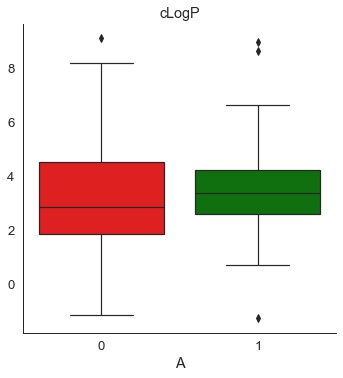


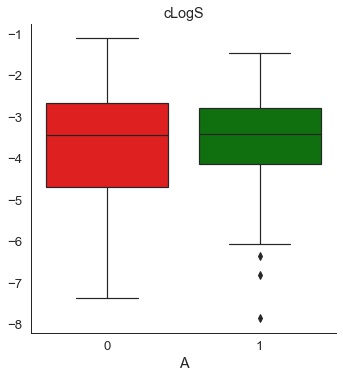

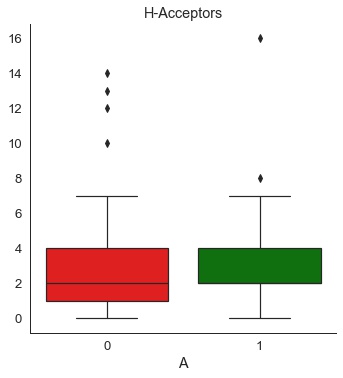


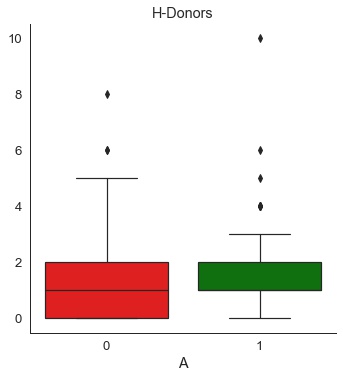

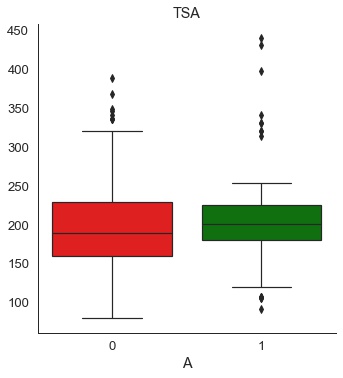


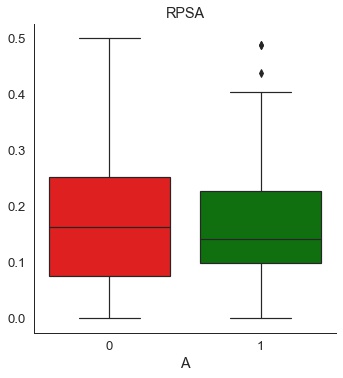

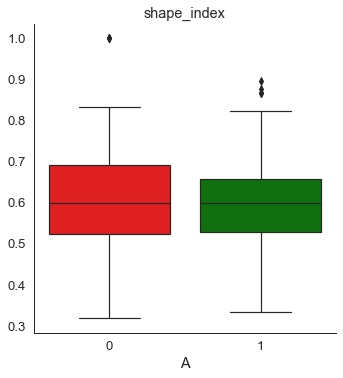


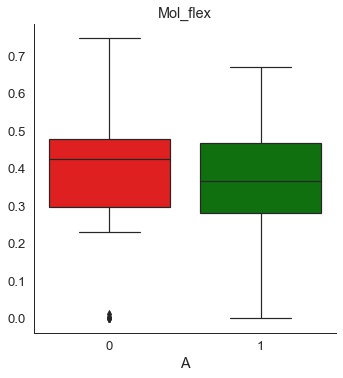

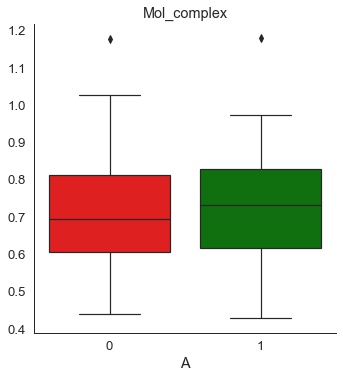


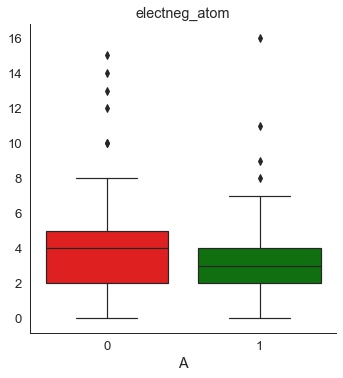

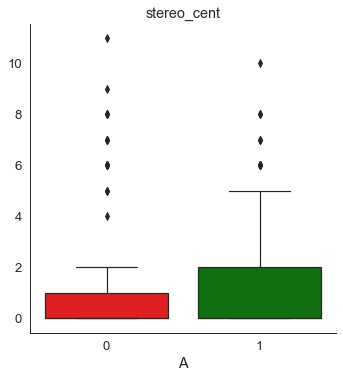


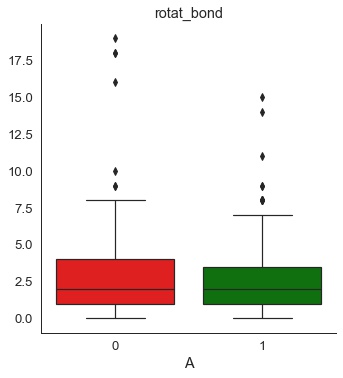

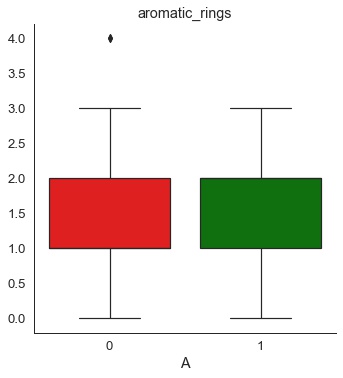


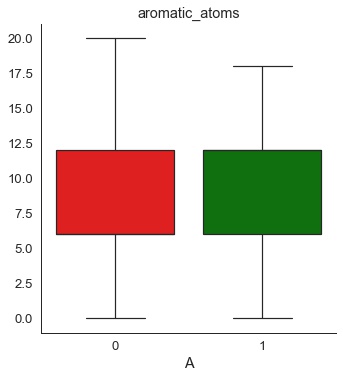


Figure S1: Boxplots representing the distribution of physiochemical descriptors computed with Datawarrior for binding compounds (B) in green and non-binding (NB) compounds in red for the EADB dataset


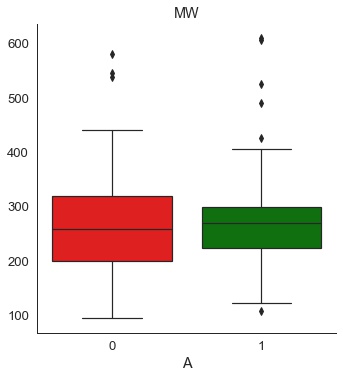

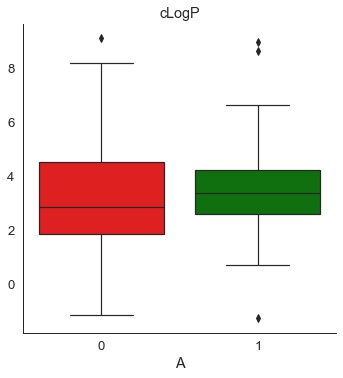

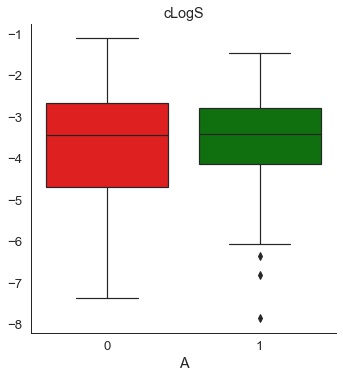

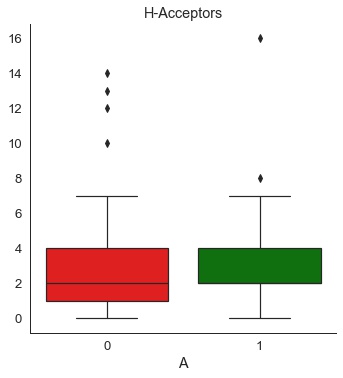

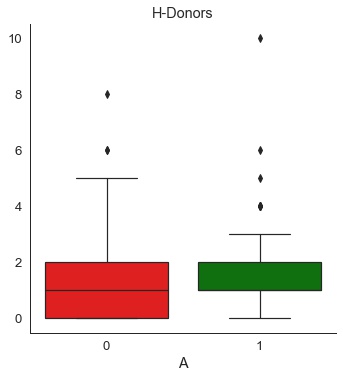

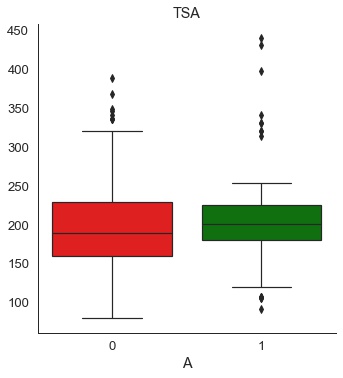

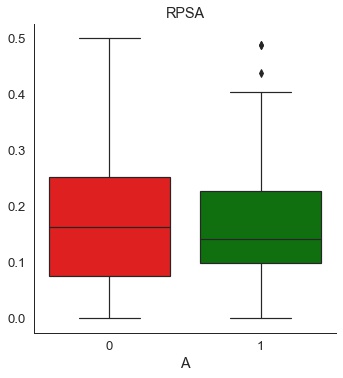

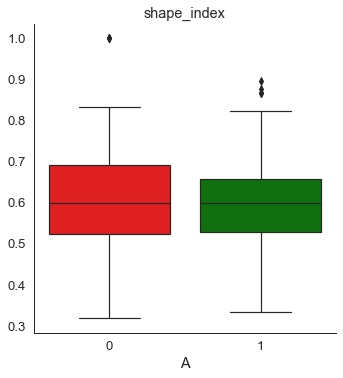

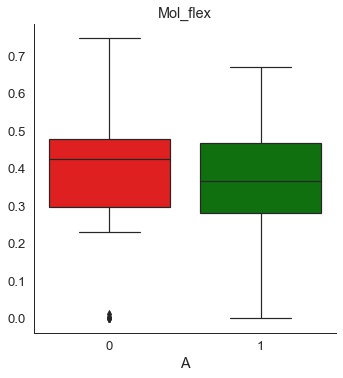

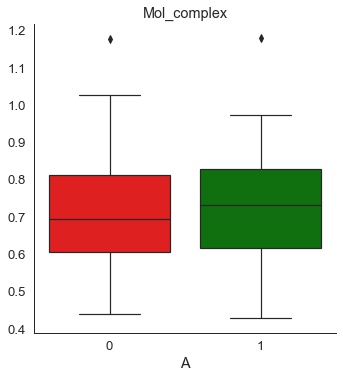

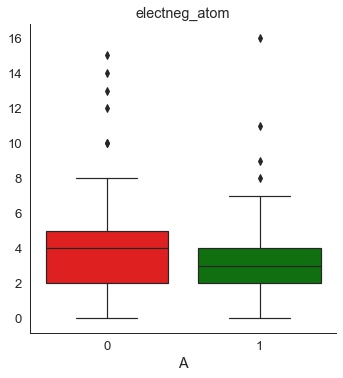

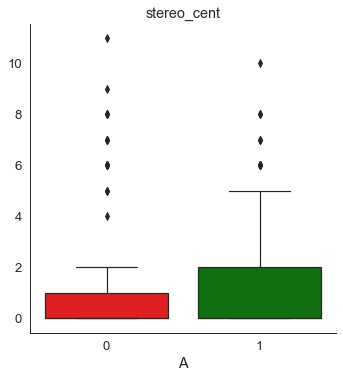

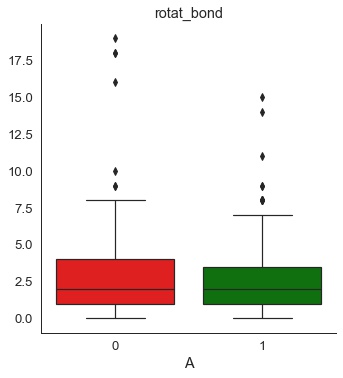

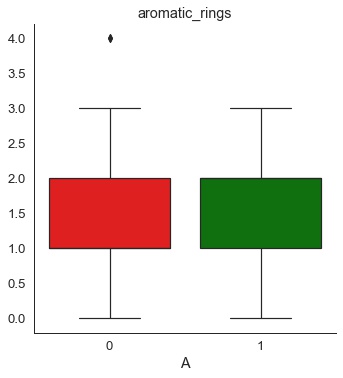

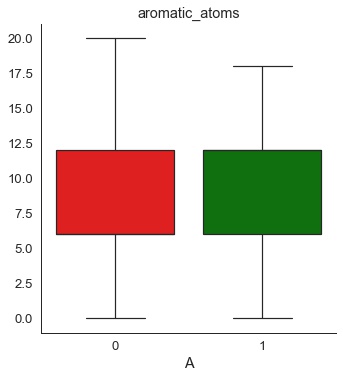


Figure S2: Boxplots representing the distribution of physiochemical descriptors computed with Datawarrior for binding compounds (B) in green and non-binding (NB) compounds in red for the NR-DBIND ERα dataset

| 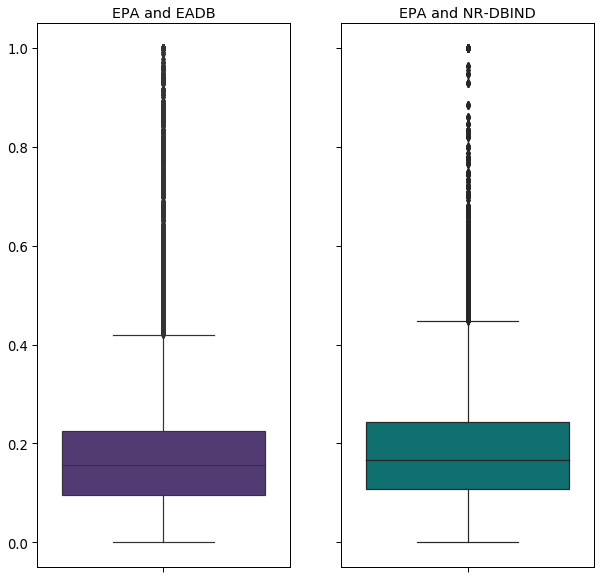 | 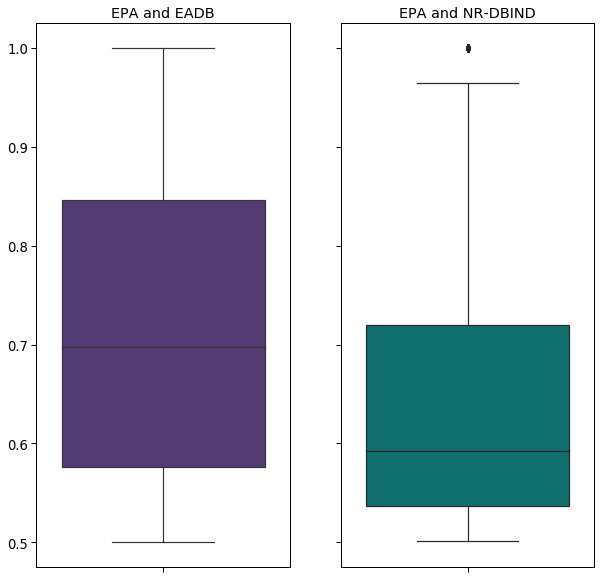 |
| --- | --- |
|  |  |

Figure S3: Boxplots representing the distribution of pairwise calculated Tanimoto coefficient between EPA database and EADB (in blue) and NR-DBIND (green) topological fingerprints. (A) Distribution for the entire database, (B) distribution of the Tc superior to 0.5

Table S1: Docking performances for both single and ensemble structure approach illustrated with the AUC of the best and the worst ensembles in terms of AUC

| SIZE | BEST | MAX_AUC | WORST | MIN_AUC |
| --- | --- | --- | --- | --- |
| Smina_Vina | | | | |
| 1 | [1a52-] | 0,70 | [2yja-] | 0,64 |
| 2 | [1qku-1a52-] | 0,70 | [2yja-1g50-] | 0,66 |
| 3 | [2yja-1qku-1a52-] | 0,70 | [2yja-1qku-1g50-] | 0,68 |
| 4 | [2yja-1x7e-1qku-1a52-] | 0,70 | [2yja-1x7e-1qku-1g50-] | 0,68 |
| 5 | [2yja-1x7e-1qku-1g50-1a52-] | 0,70 | [2yja-1xp9-1xp1-1x7e-1g50-] | 0,69 |
| 6 | [2yja-1xp1-1x7e-1qku-1g50-1a52-] | 0,70 | [2yja-1xp9-1xp1-1x7e-1qku-1g50-] | 0,69 |
| 7 | [2yja-1xp9-1xp1-1x7e-1qku-1g50-1a52-] | 0,70 | [2yja-1xp9-1xp1-1x7e-1qku-1g50-1a52-] | 0,70 |
| Smina_dkoes | | | | |
| 1 | [1qku-] | 0.708 | [1xp9-] | 0.7 |
| 2 | [2yja-1qku-] | 0.709 | [1xp9-1x7e-] | 0.70 |
| 3 | [2yja-1qku-1g50-] | 0.71 | [1xp9-1xp1-1x7e-] | 0.70 |
| 4 | [2yja-1qku-1g50-1a52-] | 0.71 | [1xp9-1xp1-1x7e-1a52-] | 0.71 |
| 5 | [2yja-1xp1-1qku-1g50-1a52-] | 0.71 | [1xp9-1xp1-1x7e-1g50-1a52-] | 0.71 |
| 6 | [2yja-1xp1-1x7e-1qku-1g50-1a52-] | 0.71 | [2yja-1xp9-1xp1-1x7e-1g50-1a52-] | 0.71 |
| 7 | [2yja-1xp9-1xp1-1x7e-1qku-1g50-1a52-] | 0.71 | [2yja-1xp9-1xp1-1x7e-1qku-1g50-1a52-] | 0.71 |
| Smina_Vinardo | | | | |
| 1 | [1a52-] | 0.68 | [2yja-] | 0.62 |
| 2 | [1xp9-1a52-] | 0.68 | [2yja-1g50-] | 0.62 |
| 3 | [1xp9-1xp1-1a52-] | 0.67 | [2yja-1qku-1g50-] | 0.62 |
| 4 | [1xp9-1xp1-1x7e-1a52-] | 0.67 | [2yja-1xp1-1x7e-1qku-] | 0.62 |
| 5 | [1xp9-1xp1-1x7e-1g50-1a52-] | 0.66 | [2yja-1xp9-1xp1-1x7e-1qku-] | 0.62 |
| 6 | [1xp9-1xp1-1x7e-1qku-1g50-1a52-] | 0.66 | [2yja-1xp9-1xp1-1x7e-1qku-1g50-] | 0.62 |
| 7 | [2yja-1xp9-1xp1-1x7e-1qku-1g50-1a52-] | 0.62 | [2yja-1xp9-1xp1-1x7e-1qku-1g50-1a52-] | 0.62 |

| Smina_ad4 | | | | |
| --- | --- | --- | --- | --- |
| 1 | [1a52-] | 0.66 | [2yja-] | 0.61 |
| 2 | [1qku-1a52-] | 0.66 | [2yja-1g50-] | 0.62 |
| 3 | [2yja-1qku-1a52-] | 0.66 | [2yja-1x7e-1g50-] | 0.64 |
| 4 | [2yja-1x7e-1qku-1a52-] | 0.66 | [2yja-1x7e-1qku-1g50-] | 0.64 |
| 5 | [2yja-1x7e-1qku-1g50-1a52-] | 0.66 | [2yja-1xp9-1xp1-1qku-1g50-] | 0.65 |
| 6 | [2yja-1xp9-1x7e-1qku-1g50-1a52-] | 0.65 | [2yja-1xp9-1xp1-1x7e-1qku-1g50-] | 0.65 |
| 7 | [2yja-1xp9-1xp1-1x7e-1qku-1g50-1a52-] | 0.65 | [2yja-1xp9-1xp1-1x7e-1qku-1g50-1a52-] | 0.65 |
| PLANTS | | | | |
| 1 | [1x7e] | 0,66 | [2yja] | 0,60 |
| 2 | [1a52-1x7e] | 0,66 | [1qku/2yja] | 0,61 |
| 3 | [1a52-1qku-1x7e] | 0,66 | [1g50/1qku/2yja] | 0,62 |
| 4 | [1a52-1g50-1qku-1x7e | 0,66 | [1qku-1xp1-1xp9-2yja] | 0,63 |
| 5 | [1a52-1g50-1qku-1x7e-1xp9 | 0,65 | [1g50-1qku-1xp1-1xp9-2yja] | 0,63 |
| 6 | [1a52-1g50-1qku-1x7e-1xp1-1xp9 | 0,65 | [1a52-1g50-1qku-1xp1-1xp9-2yja] | 0,64 |
| 7 | [1a52-1g50-1qku-1x7e-1xp1-1xp9-2yja | 0,64 | [1a52-1g50-1qku-1x7e-1xp1-1xp9-2yja] | 0,64 |
| Surflex_dock | | | | |
| 1 | [2yja | 0,45 | [1a52] | 0,40 |
| 2 | [1qku-2yja | 0,46 | [1a52-1xp1] | 0,40 |
| 3 | [1g50-1qku-2yja | 0,46 | [1a52-1xp1-1xp9] | 0,40 |
| 4 | [1g50-1qku-1xp1-2yja | 0,46 | [1a52-1x7e-1xp1-1xp9] | 0,43 |
| 5 | [1g50-1qku-1x7e-1xp1-2yja | 0,45 | [1a52-1g50-1x7e-1xp1-1xp9] | 0,43 |
| 6 | [1g50-1qku-1x7e-1xp1-1xp9-2yja | 0,45 | [1a52-1g50-1qku-1x7e-1xp1-1xp9] | 0,44 |
| 7 | [1a52-1g50-1qku-1x7e-1xp1-1xp9-2yja | 0,44 | [1a52-1g50-1qku-1x7e-1xp1-1xp9-2yja] | 0,44 |

Table S2: Maximum values of predictiveness (P(active)) associated to each scoring function and each docking approach

| **Docking approach** | **Single** | **Ensemble of 2** | **Ensemble of 3** |
| --- | --- | --- | --- |
| **Scoring function** |  |  |  |
| **Dkoes** | 0.35 | 0.35 | 0.35 |
| **Vina** | 0.151 | 0.19 | 0.223 |
| **Vinardo** | 0.115 | 0.142 | 0.155 |
| **Ad4** | 0.093 | 0.093 | 0.093 |
| **PLANTS** | 0.27 | 0.314 | 0.309 |
| **Surflex** | 0.092 | 0.137 | 0.147 |

Table S3: Sensitivity (Se), specificity (Sp), scoring threshold (TH), Enrichment factor (EF) and B-Total ratios calculated for the best scoring function (smina-dkoes and PLANTS) and corresponding to P(active)max for different docking approaches

| **Scoring function** | **Dkoes** | | | **PLANTS** | | |
| --- | --- | --- | --- | --- | --- | --- |
| **Docking approach** | **Single** | **Ensemble of 2** | **Ensemble of 3** | **Single** | **Ensemble of 2** | **Ensemble of 3** |
| **P(max)** | 0.35 | 0.35 | 0.35 | 0.27 | 0.314 | 0.309 |
| **Se** | 0.014 | 0.014 | 0.014 | 0.005 | 0 | 0 |
| **Sp** | 0.995 | 0.995 | 0.995 | 0.999 | 0.998 | 0.998 |
| **TH** | -10 | -10 | -10 | -107 | -121 | -121 |
| **EF** | 2.36 | 2.36 | 2.36 | 2.2 | 0 | 0 |
| **B-Total** | 3-14 | 3-14 | 3-14 | 1-5 | 0-5 | 0-5 |

Table S4: Pairwise RMSD computed between all the protein structures

| 1a52 | 1a52 |  |  |  |  |  |  | |
| --- | --- | --- | --- | --- | --- | --- | --- | --- |
| 1g50 | 0.640 | **1g50** |  |  |  |  |  | |
| 1qku | 0.564 | 0.383 | **1qku** |  |  |  |  |  |
| 1x7e | 0.479 | 0.551 | 0.475 | **1x7e** |  |  |  | |
| 1xp1 | 0.483 | 0.665 | 0.601 | 0.491 | **1xp1** |  |  |  |
| 1xp9 | 0.476 | 0.652 | 0.581 | 0.497 | 0.142 | **1xp9** |  | |
| 2yja | 0.435 | 0.573 | 0.506 | 0.481 | 0.411 | 0.397 | **2yja** |  |

Table S5: PDB Structures used for structure based model building. All 31 structures were used to generate SB pharmacophores and only those colored in blue were used for docking

| PDB | Exp | Resolution | Chaine | Profile | Ligand |
| --- | --- | --- | --- | --- | --- |
| 1A52 | X-ray | 2.80 | A | agonist | estradiol |
| 1ERE | X-ray | 3.10 | A | agonist | estradiol |
| 1ERR | X-ray | 2.60 | A | modulator | raloxifene |
| 1G50 | X-ray | 2.90 | A | agonist | estradiol |
| 1GWQ | X-ray | 2.45 | A | agonist | raloxifene |
| 1GWR | X-ray | 2.40 | A | agonist | estradiol |
| 1L2I | X-ray | 1.95 | A | agonist | ZINC03940885 |
| 1QKU | X-ray | 3.20 | A, B, C | agonist | estradiol |
| 1R5K | X-ray | 2.70 | A | modulator | GW5638 |
| 1SJ0 | X-ray | 1.90 | A | modulator | ZINC03949855 |
| 1X7E | X-ray | 2.80 | A, B | agonist | WAY-244 |
| 1X7R | X-ray | 2.00 | A | agonist | genistein |
| 1XP1 | X-ray | 1.80 | A | antagonist | ZINC12501375 |
| 1XP6 | X-ray | 1.70 | A | modulator | ZINC16051663 |
| 1XP9 | X-ray | 1.80 | A | antagonist | ZINC12502349 |
| 1XPC | X-ray | 1.60 | A | modulator | ZINC12502347 |
| 1YIM | X-ray | 1.90 | A | modulator | ZINC16051697 |
| 1YIN | X-ray | 2.20 | A | modulator | ZINC03948747 |
| 2BJ4 | X-ray | 2.00 | A, B | modulator | 4-hydroxytamoxifen |
| 2IOG | X-ray | 1.60 | A | modulator | ZINC16052186 |
| 2IOK | X-ray | 2.40 | A | modulator | ZINC16052185 |
| 2JF9 | X-ray | 2.10 | A, B, C | modulator | 4-hydroxytamoxifen |
| 2JFA | X-ray | 2.55 | A, B | modulator | raloxifene |
| 2OUZ | X-ray | 2.00 | A | modulator | lasofoxifene |
| 2YJA | X-ray | 1.82 | B | agonist | ESTRADIOL |
| 3DT3 | X-ray | 2.40 | A | modulator | GW368 |
| 3ERD | X-ray | 2.03 | A, B | agonist | diethylstilbestrol |
| 3ERT | X-ray | 1.90 | A | modulator | 4-hydroxytamoxifen |
| 3UUC | X-ray | 2.10 | A | antagonist | Bisphenol-C |
| 5T92 | X-ray | 2.22 | A, B | antagonist | (2E)-3-{4-[(1R)-2-(4-fluorophenyl)-6-hydroxy-1-methyl-1,2,3,4-tetrahydroisoquinolin-1-yl]phenyl}prop-2-enoic acid |
| 5T97 | X-ray | 3.00 | A, B | antagonist | (2E)-3-(4-{(1R)-6-hydroxy-1-methyl-2-[4-(propan-2-yl)phenyl]-1,2,3,4-tetrahydroisoquinolin-1-yl}phenyl)prop-2-enoic acid |
